# Iron Deficiency Without Anemia and Reduced Basal Ganglia Iron Content in Youths

**DOI:** 10.1001/jamanetworkopen.2025.16687

**Published:** 2025-06-20

**Authors:** Dimitri Fiani, Joo-won Kim, Mianzhi Hu, Ramiro Salas, Sarah Heilbronner, Jacquelyn Powers, Muhammad Haque, Stephanie Dinh, Xiaofan Huang, Darrell Worthy, Sridevi Devaraj, Junqian Xu, Chadi Calarge

**Affiliations:** 1 Baylor College of Medicine, Houston, Texas; 2 Now with Cleveland Clinic Foundation, Cleveland, Ohio; 3 Texas A&M University, College Station; 4 University of Texas Health Sciences at Houston, Houston

## Abstract

**Importance:**

Although brain iron is necessary for neurogenesis, myelination, and neurotransmitter synthesis, iron deficiency (ID) is defined solely based on hematological outcomes.

**Objective:**

To examine the association of ID without anemia with basal ganglia (BG) iron content and its structural and functional sequelae in adolescents.

**Design, Setting, and Participants:**

This cross-sectional study enrolled participants using the electronic medical record system from a large network of pediatrics clinics between December 2020 and April 2024. Otherwise healthy, unmedicated participants aged 10 to 17 years with a depressive or anxiety disorder or with no psychopathology were consecutively enrolled. Anemia and acute inflammation led to exclusion, and ID without anemia status was identified after procedure completion. Data were analyzed from May to November 2024.

**Exposure:**

Following the World Health Organization’s guidelines, ID without anemia was defined as a serum ferritin concentration less than 15 ng/mL.

**Main Outcomes and Measures:**

Participants underwent a brain magnetic resonance imaging scan to measure BG susceptibility and structures volume, a clinical interview to rate psychiatric symptoms severity, and neuropsychological testing. Multivariable regression and correlational partial least-squares analyses examined the association of ID without anemia status and BG susceptibility with each other and with BG structures volume, psychiatric symptom severity, and neuropsychological performance.

**Results:**

Among a total of 209 participants (122 [58%] female; mean [SD] age, 13.5 [2.2] years; 62 participants [30%] with ID without anemia), ID without anemia was associated with a significantly lower susceptibility in the caudate (Cohen *d* = −0.41; 95% CI, −0.72 to −0.10; *P* = .01) and putamen (*d* = −0.38; 95% CI, −0.69 to −0.07; *P* = .02), after accounting for age and sex. Notably, in females, the age by ID without anemia status had a significant 2-way interaction, indicating larger difference in caudate (β = 1.11; 95% CI, 0.08 to 2.15; *P* = .04) and putamen susceptibility (β = 0.95; 95% CI, 0.18 to 1.71; *P* = .02) with increasing age, favoring those without ID without anemia. None of the 2-way interactions were significant in males. Moreover, BG susceptibility was inversely associated with BG structures volume and psychiatric symptom severity and positively associated with neuropsychological performance, particularly in female adolescents.

**Conclusions and Relevance:**

In this cross-sectional study, ID without anemia was associated with lower striatal iron content and disrupted structure and function during adolescence, a critical period when the brain develops and accrues iron, particularly in females.

## Key Points

**Question:**

Is iron deficiency (ID) without anemia associated with basal ganglia iron content or brain structural and functional characteristics?

**Findings:**

In this cross-sectional study including 209 adolescents, ID without anemia was associated with lower caudate and putamen susceptibility, an imaging-based index of iron content, particularly in females. The association was larger with age, presumably an index of ID without anemia duration, and may have been dose dependent; basal ganglia susceptibility was associated with altered subcortical structures volume and worse psychiatric and cognitive functioning.

**Meaning:**

These findings suggest that even before anemia emerges, ID is associated with altered brain development during adolescence and adolescents at risk should be screened to optimize neuropsychiatric functioning.

## Teaser

This cross-sectional study examines the association of iron deficiency without anemia with basal ganglia iron content and its structural and functional outcomes in adolescents.

## Introduction

Despite iron’s ubiquitous role in health, iron deficiency (ID) remains the most common nutritional deficiency in the world.^1,2^ In the US, an estimated 38.6% of females aged 12 to 21 years have ID, with Black and Hispanic women being disproportionately affected.^3^ Efforts have primarily focused on screening for and treating iron deficiency anemia.^4,5,6^ However, ID without anemia is prevalent, with important clinical sequelae.^3^

The overall approach to diagnosing ID has continued to evolve (eg, ferritin model and total body iron model), given challenges in accurately capturing body iron status.^7^ However, regardless of the set of makers examined, cutoffs to establish reference ranges have focused on hematological outcomes.^3,7,8,9,10^ Even when the appropriate cutoff to diagnose ID is debated (eg, serum ferritin [sF] concentration <15 vs <25 ng/mL [to convert to micrograms per liter, multiply by 1]), the emphasis is on hemoglobin concentration or bone marrow iron content.^9,10,11,12^ This ignores the impact of ID without anemia on other vital organs, including the brain, particularly during development.^13,14^ Iron is necessary for neurogenesis, myelination, and neurotransmitter synthesis,^14^ with ID being implicated in several psychiatric and sleep disorders, as well as cognitive impairment.^15,16^

Recent advances in magnetic resonance imaging allow for indirect estimation of brain iron content. Specifically, quantitative susceptibility mapping (QSM) captures microscopic magnetic field perturbations caused by local tissue susceptibility.^17^ The paramagnetic properties of iron, present in sufficient amounts in several brain regions, particularly the basal ganglia (BG), have made it a prime focus.^17^ In fact, iron content and QSM-based susceptibility measurements are strongly correlated in gray matter structures.^18^

This cross-sectional study sought to characterize BG iron content in ID without anemia in healthy adolescents and those with depressive and anxiety disorders. In light of our previous preliminary findings,^19^ we further examined the association of iron content with BG volume, given the roles of these structures in a host of emotional and cognitive processes,^20,21^ and with psychiatric symptom severity and neuropsychological performance to emphasize potential structural and functional corelates.

## Methods

This cross-sectional study was approved by the institutional review board at Baylor College of Medicine, with written consent obtained from parents or guardians and verbal assent from participants. This study was conducted in accordance with the Strengthening the Reporting of Observational Studies in Epidemiology (STROBE) reporting guideline for cross-sectional studies.

### Participants

Unmedicated adolescents aged 10 to 17 years with a depressive or anxiety disorder or with no psychopathology were ascertained using the electronic medical record in a large pediatric health care system. Participants with anemia or other serious general medical conditions were excluded. Additional exclusion criteria are detailed in the eMethods in Supplement 1. These criteria sought to exclude participants with confounding conditions, including a history of perinatal ID. Confirming eligibility was first based on medical record review followed by querying the parent or guardian (eFigure 1 in Supplement 1). Eligible participants were consecutively enrolled. Race and ethnicity were self-reported. In addition to Black and Hispanic, people could identify as American Indian or Alaskan Native, Asian, Caucasian, Native Hawaiian or Other Pacific Islander, more than 1 race, and unknown or not reported. Race and ethnicity data were assessed given the difference in the prevalence of ID across racial and ethnic groups.

### Procedures

At a single visit, participants, parents or guardians, and the study psychiatrist completed rating scales (eMethods in Supplement 1). Moreover, the psychiatrist established any psychiatric diagnoses.^22^

A blood sample was obtained to measure hemoglobin (HemoCue Hb 201+, HemoCue AB); sF, the most clinically used marker of body iron status; and C-reactive protein, to rule out acute inflammation, which can result in elevated sF.^10^ Both markers were assayed in a clinically certified laboratory using Vitros 5600 and 7600 Chemistry Systems (Ortho Clinical Diagnostics). Importantly, sF is not routinely measured as part of standard clinical care; thus, ID without anemia status was determined after study procedures completion.

### Brain Imaging

A 3T Achieva scanner (Philips) was used to acquire the QSM and T1-weighted images. Technical details are provided in the eMethods in Supplement 1.

### Statistical Analysis

Participants with C-reactive protein concentrations greater than 0.50 mg/dL (to convert to milligrams per liter, multiply by 10), indicating acute inflammation,^10^ were excluded from analyses related to sF. Following the World Health Organization guidelines, ID was defined as sF less than 15 ng/mL.^10^ Group differences between participants with and without ID without anemia were compared using *t* test or Wilcoxon rank sum test for continuous variables and χ^2^ or Fisher Exact test for categorical variables.

The primary outcome of this study was BG iron content, while secondary outcomes included subcortical structure, psychiatric symptom severity, and neuropsychological performance. Two analytical approaches were used to test our hypotheses, a targeted one and an agnostic one. The targeted approach used multivariable regression analysis, accounting for age and sex. This was chosen to allow using ID without anemia status as a categorical variable of interest, as it is biologically implausible that brain iron content readily fluctuates with body iron content. To minimize the risk of type I error, only selected comparisons were carried out, with the left and right brain structures’ metrics combined. Given their large number (eMethods in Supplement 1), the neuropsychological variables were excluded from the targeted approach, as we did not have an a priori hypothesis involving any one of the tasks. Cohen *d* effect sizes were computed.^23^ For ease of interpretation, where applicable, a median split was used to divide the participants based on the susceptibility values in the subcortical regions of interest.

The second approach used correlational partial least squares (PLS) regression analysis to accommodate the large number of available variables, across the susceptibility, anatomical, psychiatric, and neuropsychological domains (eMethods and eResults in Supplement 1).^24^ The PLS approach allows examining the left and right structures separately but does not accommodate categorical variables (ie, ID without anemia status).

A sample size of 210 participants allows the detection of a small to medium effect size (*d* = 0.39) for the difference between participants with and without ID without anemia. *P* values were 2-sided, and statistical significance was set at *P* < .05. Analyses used SAS software version 9.4 (SAS Institute). Data were analyzed from May to November 2024.

## Results

### Participants

Between December 2020 and April 2024, 240 participants were enrolled, with 31 excluded for failing to meet the inclusion and exclusion criteria or having poor-quality magnetic resonance imaging scans. The final sample included 209 participants (122 [58%] female; mean [SD] age, 13.5 [2.2] years; 62 participants [30%] with ID without anemia). An additional 5 participants were excluded from the analyses involving sF either due to missing sF value (1 participant) or elevated C-reactive protein concentration (4 participants).

Compared with the group with sF 15 ng/mL or greater, the ID without anemia group included more participants who self-identified as female, especially postmenarche, and as Black or Hispanic (Table 1). This group also had more severe anxiety symptoms, smaller brain volumes, and lower caudate susceptibility values (Table 1).

**Table 1.** Demographic and Clinical Characteristics of the Overall Cohort and by Iron Status

Characteristic	Individuals, No. (%)			*P* value
	Overall (N = 209)	ID without anemia (n = 62)^a^	No ID (n = 147)	
Sex				
Male	87 (42)	17 (20)	70 (80)	.007
Female	122 (58)	45 (73)	77 (52)	
Age, mean (SD), y	13.5 (2.2)	13.7 (2.0)	13.4 (2.3)	.36
Tanner stage				
1	15 (7)	3 (5)	12 (8)	.41
2	38 (18)	8 (13)	30 (20)	
3	44 (21)	13 (21)	31 (21)	
4	70 (33)	26 (42)	44 (29)	
5	42 (20)	12 (19)	30 (20)	
Race and ethnicity				
Black or Hispanic	115 (55)	41 (66)	74 (50)	.04
Other^b^	94 (45)	21 (34)	83 (50)	
BMI Z-score, mean (SD)^c^	0.43 (0.76)	0.55 (0.69)	0.35 (0.77)	.08
Postmenarche	79 (67)	35 (80)	44 (59)	.03
Age at menarche, mean (SD), y	11.4 (1.2)	11.5 (1.3)	11.3 (1.2)	.69
Time since menarche, mean (SD), y	3.0 (1.7)	3.1 (1.7)	2.8 (1.7)	.42
Hemoglobin, mean (SD), g/dL	13.8 (1.2)	13.3 (1.0)	14.1 (1.1)	<.001
sF, mean (SD), ng/m:	24.7 (17.3)	10.0 (2.9)	30.9 (17.1)	<.001
Susceptibility values based on quantitative susceptibility mapping, mean (SD), ppb				
Caudate	25.5 (5.9)	24.0 (5.7)	26.1 (5.5)	.02
Putamen	19.5 (5.2)	18.8 (5.0)	19.8 (5.3)	.23
Pallidum	113.9 (14.8)	114.6 (12.5)	113.7 (15.6)	.69
Brain volumetric data, mean (SD), mm^3^				
Caudate	7726 (895)	7484 (852)	7829 (896)	.01
Putamen	10 613 (1162)	10 190 (1101)	10 794 (1144)	<.001
Pallidum	3684 (427)	3520 (362)	3755 (435)	<.001
Intracranial volume, ×10^6^ mm^3^	1.489 (0.164)	1.441 (0.171)	1.510 (0.157)	.006
Psychiatric characteristics				
Anxiety disorders^d^	109 (52)	40 (65)	69 (47)	.02
Depressive disorders^e^	58 (28)	20 (32)	38 (26)	.35
Externalizing disorders^f^	16 (8)	5 (8)	11 (7)	.89
Other disorders^g^	10 (5)	2 (3)	8 (5)	.49
CDRS-R T Score, mean (SD)	50.1 (14.2)	52.0 (12.8)	49.2 (14.7)	.20
PARS Score, mean (SD)	12.0 (8.1)	13.6 (7.5)	11.3 (8.2)	.06

Abbreviations: BMI, body mass index; CDRS-R, Children’s Depression Rating Scale – Revised; ID, iron deficiency; PARS, Pediatric Anxiety Rating Scale; ppb, parts per billion; sF, serum ferritin.

SI conversion factors: To convert hemoglobin to grams per liter, multiply by 10; sF to micrograms per liter, multiply by 1.

^a^ AD without anemia was defined as sF less than 15 ng/mL.

^b^ Other includes non-Hispanic Asian and White participants, which were combined given their lower propensity to have ID compared with Black and Hispanic populations.

^c^ Z scores were age- and sex-specific.

^d^ Including generalized anxiety disorder, separation anxiety disorder, social anxiety disorder, panic disorder, and specific phobia.

^e^ Including major, persistent, and other specified depressive disorders.

^f^ Including attention deficit hyperactivity disorder and oppositional defiant disorder.

^g^ Including obsessive compulsive disorder, posttraumatic stress disorder, and adjustment disorder.

After adjusting for age (β = 0.12; 95% CI, 0.06 to 0.19; *P* < .001) and sex (β = 0.80; 95% CI, 0.51 to 1.09, *P* < .001), sF was positively associated with hemoglobin concentration (β = 0.01; 95% CI, 0.00 to 0.02; *P* = .004), which was 0.69 g/dL (to convert to grams per liter, multiply by 10) lower in participants with ID without anemia (Cohen d = −0.71; 95% CI, −1.01 to −0.41; *P* < .001).

### Association Between Body Iron Status and Brain Susceptibility

After controlling for age and sex, hemoglobin concentration was positively associated with the susceptibility values in the putamen and pallidum, whereas having ID without anemia (ie, sF <15 ng/mL) was associated with lower susceptibility in the caudate (*d* = −0.41; 95% CI, −0.72 to −0.10; *P* = .01) and putamen (*d* = −0.38; 95% CI, −0.69 to −0.07; *P* = .02) (Table 2). After adjusting for age and sex, participants with ID without anemia were twice as likely to have a caudate susceptibility value below the median (odds ratio, 2.1; 95% CI, 1.1 to 4.0; *P* = .03).

**Table 2.** Association Between Basal Ganglia Susceptibility and Body Iron

Measure	Caudate susceptibility, ppb		Putamen susceptibility, ppb		Pallidum susceptibility, ppb	
	Regression estimate (95% CI)^a^	*P* value	Regression estimate (95% CI)^a^	*P* value	Regression estimate (95% CI)^a^	*P* value
Hemoglobin, g/dl	−0.16 (−0.93 to 0.62)	.69	0.69 (0.08 to 1.31)	.03	1.94 (0.17 to 3.72)	.03
sF, ng/mL	0.02 (−0.02 to 0.07)	.33	0.01 (−0.02 to 0.05)	.48	−0.06 (−0.17 to 0.05)	.29
ID without anemia present^b^	−2.29 (−4.02 to −0.55)	.01	−1.69 (−3.05 to −0.32)	.02	−0.64 (−4.77 to 3.50)	.76

Abbreviations: ID, iron deficiency; ppb, parts per billion; sF, serum ferritin.

SI conversion factors: To convert hemoglobin to grams per liter, multiply by 10; serum ferritin to micrograms per liter, multiply by 1.

^a^ Regression estimates of the association with body iron-related variables. The models adjusted for age and sex.

^b^ Defined as sF concentration less than 15 ng/mL.

**Figure 1.**
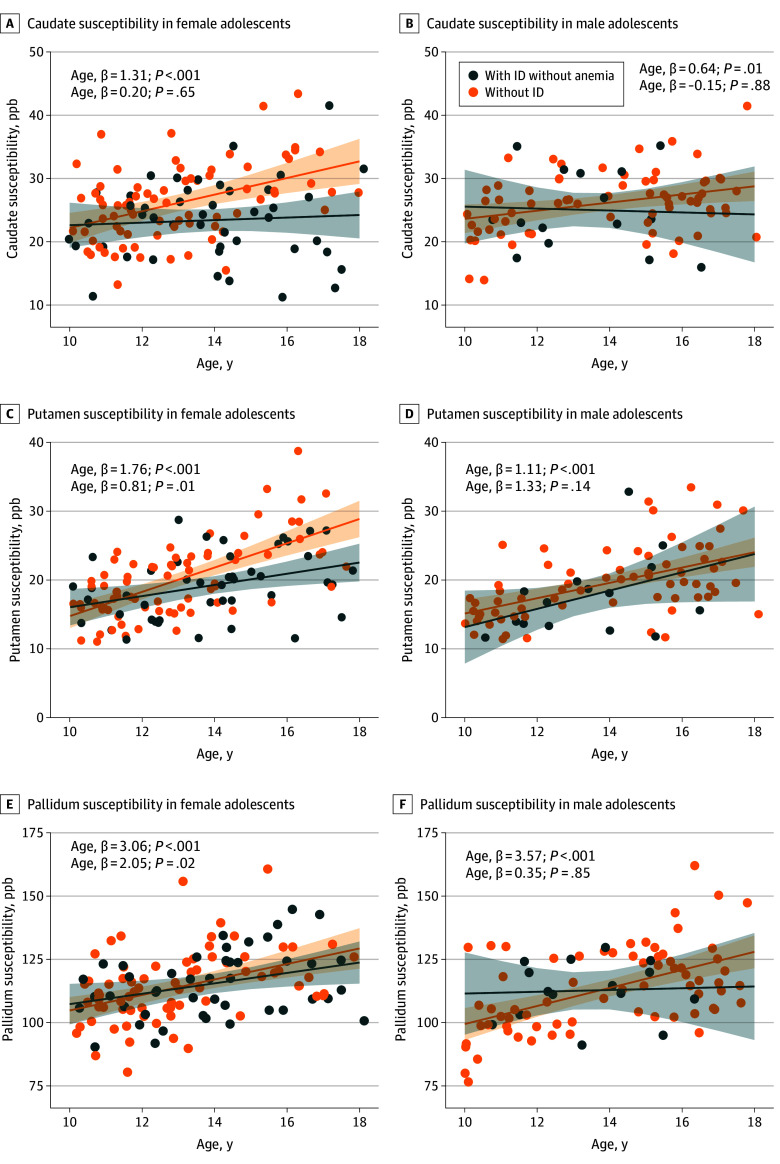
Association Between Basal Ganglia Susceptibility and Age, by Iron Status and Sex Association between susceptibility (parts per billion [ppb], a magnetic imaging resonance-based marker highly correlated with iron content) in the basal ganglia and age across participants with iron deficiency (ID) without anemia and those without ID, in female and male participants. The lines represent the regression lines estimating basal ganglia susceptibility from models including age, iron deficiency without anemia status, and their interaction, with the shaded area representing the 95% CIs. As can be seen, female participants with ID without anemia did not show the expected higher caudate and putamen iron content with increasing age observed in female participants without ID.

In female participants, the age by ID without anemia status 2-way interaction was significant in the models estimating caudate (β = 1.11; 95% CI, 0.08 to 2.15; *P* = .04) and putamen (β = 0.95; 95% CI, 0.18 to 1.71; *P* = .02) susceptibility (Figure 1A and C) but not in the model estimating pallidum susceptibility (Figure 1E). In contrast, none of the 2-way interactions were significant in males (Figure 1B, D, and F). The quadratic effect of age was not significant.

**Figure 2.**
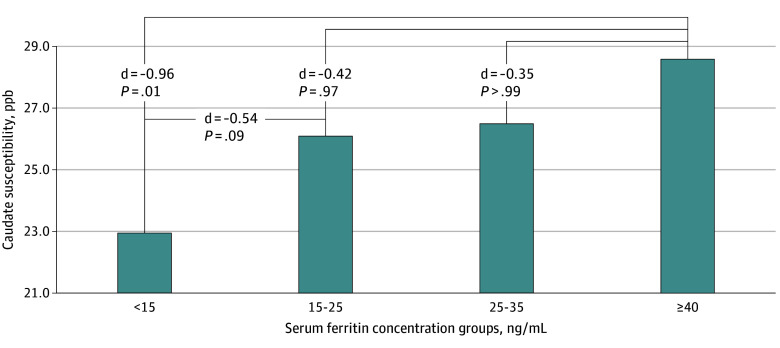
Caudate Susceptibility Across Serum Ferritin Concentration Subgroups in Female Adolescents After Adjusting for Age *P* values are Bonferonni corrected. ppb indicates parts per billion.

Caudate susceptibility was compared in female adolescents with sF less than 15 ng/mL, 15 to less than 25 ng/mL, 25 to less than 35 ng/mL, and 40 ng/mL or greater to examine the presence of a dose-dependent association. After adjusting for age, sF group status was associated with caudate susceptibility (*P* = .009). Following Bonferroni correction, post hoc analyses showed a dose-dependent association, with the largest difference being between females with ID without anemia and those with sF of 40 ng/mL or greater (*d* = −0.96; 95% CI, −1.55 to −0.37; *P* = .01) (Figure 2).

### Association of Body Iron Status With Brain Susceptibility and Brain Structure

After adjusting for age, sex, and estimated total intracranial volume (eTIV), having ID without anemia was associated with larger pallidum volume (*d* = 0.43; 95% CI, 0.12 to 0.74; *P* = .007) in the sample overall and with larger palladium and putamen volumes in female participants (pallidum: *d* = 0.50; 95% CI, 0.12 to 0.89; *P* = .01; putamen: *d* = 0.54; 95% CI, 0.16 to 0.93; *P* = .006) (Table 3). We did not find any significant associations of body iron status with eTIV, with caudate volume, or in male participants (Table 3).

**Table 3.** Associations Between Iron Deficiency Status and Basal Ganglia Susceptibility (Divided Based on the Median Split) and Brain Structures Volume

Group	eTIV^a^^,^^b^			Caudate volume, mm^3^^a^^,^^c^			Putamen volume, mm^3^^a^^,^^c^			Pallidum volume, mm^3^^a^^,^^c^		
	Regression estimate (95% CI)	Cohen *d* (95% CI)	*P* value	Regression estimate (95% CI)	Cohen *d* (95% CI)	*P* value	Regression estimate (95% CI)	Cohen *d* (95% CI)	*P* value	Regression estimate (95% CI)	Cohen *d* (95% CI)	*P* value
**ID without anemia present** ^d^												
Total	30 290.0 (−12 037.6 to 72 617.5)	0.22 (−0.09 to 0.53)	.16	161.8 (−69.6 to 393.2)	0.22 (−0.09 to 0.52)	.17	284.5 (−2.3 to 571.3)	0.31 (0 to 0.61)	.05	142.3 (40.7 to 243.8)	0.43 (0.12 to 0.74)	.006
Females	25 694.6 (−25 685.8 to 77 075.0)	0.19 (−0.19 to 0.57)	.32	197.6 (−89.1 to 484.3)	0.26 (−0.12 to 0.65)	.17	485.6 (143.3 to 827.8)	0.54 (0.16 to 0.93)	<.001	165.1 (39.2 to 291.0)	0.50 (0.12 to 0.89)	.01
Males	31 743.6 (−46 440.9 to 109 928.1)	0.22 (−0.32 to 0.76)	.42	268.4 (−115.5 to 652.4)	0.38 (−0.16 to 0.92)	.17	−27.6 (−558.7 to 503.5)	−0.03 (−0.57 to 0.51)	.92	117.0 (−62.2 to 296.2)	0.35 (−0.19 to 0.90)	.20
**Caudate susceptibility, ppb**												
Total	41 701.0 (3503.9 to 79 898.0)	0.31 (0.03 to 0.58)	.03	−115.3 (−326.8 to 96.3)	−0.15 (−0.44 to 0.13)	.28	−249.3 (−513.9 to 15.4)	−0.27 (−0.55 to 0.02)	.06	22.9 (−72.2 to 118.1)	0.07 (−0.21 to 0.35)	.64
Females	36 444.3 (−12 384.4 to 85 273)	0.28 (−0.09 to 0.64)	.14	−50.4 (−328.6 to 227.8)	−0.07 (−0.44 to 0.3)	.72	−370.9 (−706.8 to −35.1)	−0.41 (−0.78 to −0.04)	.03	33.9 (−91.5 to 159.3)	0.10 (−0.27 to 0.47)	.59
Males	50 723.4 (−11 706.1 to 11 3152.8)	0.35 (−0.08 to 0.79)	.11	−251.9 (−560.0 to 56.2)	−0.36 (−0.8 to 0.08)	.11	−93.3 (−531 to 344.4)	−0.09 (−0.54 to 0.35)	.67	2.9 (−145.5 to 151.2)	0.01 (−0.43 to 0.45)	.97
**Putamen susceptibility, ppb**												
Total	47 494.7 (4929.6 to 90 059.9)	0.35 (0.04 to 0.66)	.03	−53.8 (−290.3 to 182.7)	−0.07 (−0.39 to 0.24)	.65	−450.5 (−741.7 to −159.4)	−0.49 (−0.80 to −0.17)	.003	−106.0 (−211.2 to −0.8)	−0.32 (−0.63 to 0.00)	.048
Females	52 892 (−1496.0 to 107 280.1)	0.40 (−0.01 to 0.82)	.06	11.9 (−302 to 325.8)	0.02 (−0.4 to 0.44)	.94	−212.4 (−596.7 to 172.0)	−0.23 (−0.65 to 0.19)	.28	3.5 (−138.0 to 145.1)	0.01 (−0.41 to 0.43)	.96
Males	42 515.8 (−27 102.9 to 112 134.4)	0.29 (−0.19 to 0.78)	.23	−170.4 (−512.8 to 172.1)	−0.24 (−0.73 to 0.24)	.33	−787.8 (−1238.4 to −337.3)	−0.85 (−1.34 to −0.36)	<.001	−249.6 (−403.5 to −95.7)	−0.79 (−1.28 to −0.30)	.002
**Pallidum susceptibility, ppb**												
Total	12 980.7 (−26 832.6 to 52 794.0)	0.09 (−0.19 to 0.38)	.52	−61.4 (−277.9 to 155.1)	−0.08 (−0.37 to 0.21)	.58	−80.4 (−352.7 to 192.0)	−0.09 (−0.37 to 0.20)	.56	−65.8 (−162.6 to 31.0)	−0.20 (−0.49 to 0.09)	.18
Females	−20 337.8 (−69 890.1 to 29 214.5)	−0.15 (−0.52 to 0.22)	.42	13.1 (−265.8 to 292.1)	0.02 (−0.36 to 0.39)	.93	−74.6 (−417.6 to 268.4)	−0.08 (−0.45 to 0.29)	.67	−68.5 (−193.7 to 56.6)	−0.20 (−0.58 to 0.17)	.28
Males	62 824.6 (−3456.1 to 129 105.3)	0.44 (−0.02 to 0.91)	.06	−240.7 (−572.5 to 91.1)	−0.34 (−0.82 to 0.13)	.15	−85.3 (−555.2 to 384.6)	−0.09 (−0.56 to 0.39)	.72	−94.6 (−252.5 to 63.3)	−0.28 (−0.76 to 0.19)	.24

Abbreviations: eTIV, estimated total intracranial volume; ID, iron deficiency; ppb, parts per billion.

^a^ Regression estimates associated with each variable (ie, ID without anemia status and caudate, putamen, and pallidum susceptibility status based on a median split), followed by effect size (Cohen d).

^b^ Models estimating eTIV accounted for age and sex.

^c^ Models estimating basal ganglia volumes additionally accounted for eTIV.

^d^ Defined as serum ferritin concentration less than 15 ng/mL (to convert to micrograms per liter, multiply by 1).

After adjusting for age and sex, caudate (*d* = 0.31; 95% CI, 0.03 to 0.58; *P* = .04) and putamen (*d* = 0.35; 95% CI, 0.04 to 0.66; *P* = .03) susceptibility were positively associated with eTIV (Table 3). After adjusting for age and eTIV, female participants in the upper median for caudate susceptibility had significantly smaller putamen volumes (d = −0.41; 95% CI, −0.78 to −0.04; *P* = .03), while males in the upper median for putamen susceptibility had smaller putamen (*d* = −0.85; 95% CI, −1.34 to −0.36; *P* < .001) and pallidum (*d* = −0.79; 95% CI, −1.28 to −0.30; *P* = .002) volumes (Table 3).

### Association of Body Iron Status With Brain Susceptibility and Psychopathology

After controlling for age, sex, and clinical group, the presence of ID without anemia was not associated with the Children’s Depression Rating Scale-Revised T-score or with the Pediatric Anxiety Rating Scale score. In contrast, having a caudate susceptibility value above the median was associated with a lower depression score in all participants (*d* = −0.34; 95% CI, −0.62 to −0.06; *P* = .02) and with a lower anxiety score in participants with a depressive or anxiety disorder (2-way interaction: d = −0.54; 95% CI, −1.03 to −0.04; *P* = .01). Putamen and pallidum susceptibility was not associated with either symptom rating scale score.

## Discussion

To our knowledge, this cross-sectional study is the first study to show that BG iron content was reduced in adolescents with ID without anemia. This deficit had greater magnitude with older age, particularly in female adolescents. We also found brain structural and functional associations, highlighting the health impact of ID without anemia and, consequently, the urgent need for further confirmatory research.

Iron rapidly accrues in the brain through young adulthood,^13,25,26,27^ where it is incorporated into various structural and transport proteins, serves as a cofactor for many essential enzymes, and is critical for neurogenesis, myelin formation, and neurotransmitter production, particularly dopamine.^28,29,30,31^ The role of iron in neuropsychiatric performance has long been recognized, with ID disrupting and iron repletion restoring psychiatric and cognitive functioning.^15,16,25,28,30,32^ However, ID remains common, including in adolescents, due to their accelerated physical growth and iron loss (eg, from menstruation), resulting in a misalignment between body requirements and dietary intake.^3,33^

Despite the high prevalence of ID without anemia and its impact on physical, emotional, and cognitive functioning,^3,15,16,19,34^ screening guidelines are lacking. Compared with ID with anemia, screening for ID without anemia is more challenging for several reasons, including the absence of consensus around the threshold for diagnosis.^7,8,35^ Definitions that require abnormalities across several biological markers are too restrictive.^8^ Even definitions based solely on sF have included a wide range of proposed normal sF concentrations.^3,9,10,15,16,19,36^ In this study, we used a conservative cutoff for sF to avoid controversy and cast light on its sequelae, hoping to trigger a call for universal screening in all at-risk age groups.

Brain iron turnover has been reported as very slow,^37^ implying that brain iron may be shielded in ID. However, increasing evidence suggests otherwise. In a widely cited postmortem study, BG iron content was substantially reduced in 3 individuals with large hemorrhages.^13^ In neonates, brain iron concentration was reduced once 75% of hepatic iron stores were depleted.^38^ Moreover, hemoglobin concentration was associated with brain susceptibility in middle-aged and older adults.^39^ Iron supplementation improves cognitive and psychiatric symptoms in patients with ID without anemia, whereas the iron chelator deferiprone reduces hippocampal brain susceptibility in older adults.^15,40,41^ This evidence, combined and consistent with our findings, suggests that the brain may be vulnerable to ID, even prior to the emergence of anemia. Given the brain’s vital function, it is likely that brain iron turnover is tightly regulated within a certain physiological range, similar to other processes (eg, intracranial pressure),^42^ rather than allowing for daily fluctuations related to circulating iron. It is more biologically plausible that brain iron content would be affected only when the brain’s homeostatic mechanisms become overwhelmed. This may be particularly true in states of ID, when iron is no longer available to access the brain, vs states of iron overload,^43^ given excess iron’s potential to cause neurotoxic effects.

A related notable observation is that BG susceptibility was associated with hemoglobin concentration and ID without anemia status, but not with sF (as a continuous variable). Again, this is likely due to the underlying physiology. While sF less than 15 ng/mL is a marker of bone marrow iron depletion, it is not an indicator of its severity.^10^ In contrast, sF 15 ng/mL or greater does not necessarily rule in or out ID without anemia, particularly the range in which most of our participants fell.^35^ Conversely, in the absence of other etiologies, a reduction in hemoglobin concentration reflects sufficiently depleted bone marrow iron.^7,8^ This aligns with our findings of lower hemoglobin concentration in participants with ID without anemia. Of course, the caveat with using hemoglobin as the marker of interest is that brain iron would have long been diverted by the time hematopoiesis is impacted.^44^

Our findings have important clinical significance, highlighting an urgent need for further investigations. During a critical period of brain iron accrual and brain development,^13,45^ ID without anemia was associated with lower BG susceptibility, consistent with reduced brain iron content. This association was dependent on the severity of ID and had greater magnitude with age in female adolescents. Our study’s cross-sectional design precludes determining the age of onset of ID. However, a recent study found that ID persisted 3 years later in most individuals.^46^ Because of the growth spurt and menarche during adolescence, it is likely that our female participants experienced ID for years prior to study enrollment. Notably, the ID status by age slope associated with caudate and putamen susceptibility in female participants with ID without anemia vs those without ID intersected between ages 10 and 11 years, which occur during the period of accelerated height growth for most individuals and shortly before the average age of menarche.^47^ In contrast, the same slopes, albeit not statistically significant, intersected approximately 2 to 3 years later in boys, generally equivalent to the lag between girls and boys in starting pubertal development.^47^ This raises the question of whether boys with ID without anemia will show a similar reduction in dorsal striatal (ie, caudate and putamen) susceptibility, with increasing magnitude with age, if assessed long enough after the onset of their puberty. Our study could not fully address this question, given the limited number of boys with ID without anemia, with the oldest being aged approximately 16 years. Importantly, the fact that both the caudate and putamen showed comparable patterns of ID without anemia-moderated change in susceptibility with age adds validity to the findings, given that these 2 nuclei originate from the same embryological precursor structure and are key nodes in the cortico-striatal circuits.^48^ In contrast, ID without anemia was not associated with pallidum iron content, perhaps because iron accrual in the pallidum is more resilient to ID without anemia.

While it is notable that ID without anemia was associated with lower dorsal striatal susceptibility, it is also clinically significant that the latter is associated with structural and functional alterations. Susceptibility in the BG was inversely associated with the volume of several subcortical structures. Change in BG volume during late childhood and adolescence follows an inverted U-shaped trajectory, with different tempos in boys and girls.^45^ Our observations suggest that iron content may moderate this developmental process, perhaps by altering myelination or pruning. This may also result from impaired dopaminergic signaling, consistently reported in animal models of ID.^49^ Additionally, increased BG volumes in the context of altered dopaminergic signaling has been reported following exposure to psychostimulants and treatment with antipsychotic medications with potent dopamine D_2_ receptor antagonist activity.^50,51^ This association was more significant in female than male participants, again, likely reflecting differences in the age of onset of these developmental changes and duration of ID without anemia.^45^

The alteration in dorsal striatal susceptibility was also associated with more severe anxiety and depressive symptoms and impairment in neuropsychological functioning, highlighting the clinical implications of ID without anemia. Of note, not all measures of psychopathology and cognitive functioning showed associations with body iron content and dorsal striatal susceptibility. This apparent inconsistency likely reflects differences in ID onset in relation to each participant’s development and the fact that different affective and neuropsychological processes implicate different parts of these complex pathways.^14,45^ While it is not likely that ID without anemia by itself is a cause of mental illness, disruption to normal brain development, including the establishment of interconnected brain networks,^48^ may be an important factor in predisposing, precipitating, or even perpetuating psychopathology in at-risk individuals by interfering with neuropsychological development.

### Limitations

This study has generated critical findings to inform public health policy, yet several limitations must be noted. First, the cross-sectional design precludes determining the age of onset of ID without anemia. While we thoroughly screened for risk factors associated with ID in utero and in infancy, this could not be fully ruled out, which is important, given that early-life ID may have long-term sequelae.^14^ Future studies should follow youth longitudinally, starting before ID emerges, and enroll more male participants with ID without anemia, as their small number in our study likely limited statistical power. While iron is thought to be the main contributor to the susceptibility measurement in the brain, given its availability at a sufficient concentration and its paramagnetic property, other elements may influence susceptibility and some molecules, including myelin, attenuate it, due to their diamagnetic properties.^17^ Future research should seek to disentangle the contribution of iron accrual from that of myelin.^17^ ID without anemia was defined using the most conservative cutoff for a single marker, sF.^10^ While lower sF cutoffs maximize the specificity of the test for ID, its sensitivity suffers.^8,9^ Future studies should examine how alternative sF cutoffs or additional markers, eg, soluble transferrin receptor, may augment our findings, particularly that the association between ID without anemia and striatal susceptibility may be dose-dependent. Although our primary analytical approach did not adjust for the number of secondary comparisons carried out, given the narrow focus of the analyses, the broader PLS analyses minimize the risk of type 1 error. In fact, the overall convergence in the results between these 2 approaches adds rigor to the findings. Finally, this study focused on the BG because they have the highest iron content in the brain and are key structures in cortico-striatal networks.^13,48^ Future studies should examine other brain regions.

## Conclusions

To our knowledge, this cross-sectional study is the first to show that ID without anemia in adolescents was associated with reduced dorsal striatal susceptibility, reflecting reduced iron content, specifically in girls. The findings further suggest that the magnitude of this outcome was dose dependent and increased over time, presumably with more extended exposure to ID, and that a range of clinically relevant correlates emerged, including alteration in brain structure, cognitive function, and psychiatric symptom severity. This calls for a consideration to update practice guidelines to more aggressively identify and address ID without anemia, particularly that minoritized racial and ethnic groups are disproportionately affected.

## Notes

Supplement 1.
eMethods.

eResults.
**eTable 1.** Additional Clinical Characteristics of the Participants as a Group and Divided Based on Iron Deficiency Status **eTable 2.** Results of the Multivariable Regression Analyses Examining the Associations Between Age, Iron Deficiency Status, and Their Interaction and Basal Ganglia Susceptibility **eTable 3.** Strength of the Associations Between Basal Ganglia Susceptibility Values and Structural and Cognitive Outcomes Emerging from the Partial Least Squares Analyses **eFigure 1.** Diagram Describing Participant Ascertainment and Enrollment **eFigure 2.** Sample Distribution of Susceptibility Across the Caudate, Putamen, and Globus Pallidum, in Female and Male Adolescents **eFigure 3.** Overall Results of the Partial Least Squares Analyses Examining the Associations Between Markers of Body Iron Status and Subcortical Structure Volumes, Psychopathology, and Neuropsychological Task Performance **eFigure 4.** Overall Results of the Partial Least Squares Analyses Examining the Associations Between Basal Ganglia Susceptibility Values and Subcortical Structure Volumes, Psychopathology, and Neuropsychological Task Performance
eReferences.

Supplement 2.
Data Sharing Statement
