# Supplementary material for: Iron Deficiency Without Anemia and Reduced Basal Ganglia Iron Content in Youths
Source: JAMA Netw Open. 2025 Jun 20;8(6):e2516687. doi: 10.1001/jamanetworkopen.2025.16687 (PMC12181794; doi:10.1001/jamanetworkopen.2025.16687)
Supplement: Supplement 1. — eMethods. eResults. eTable 1. Additional Clinical Characteristics of the Participants as a Group and Divided Based on Iron Deficiency Status eTable 2. Results of the Multivariable Regression Analyses Examining the Associations Between Age, Iron Deficiency Status, and Their Interaction and Basal Ganglia Susceptibility eTable 3. Strength of the Associations Between Basal Ganglia Susceptibility Values and Structural and Cognitive Outcomes Emerging from the Partial Least Squares Analyses eFigure 1. Diagram Describing Participant Ascertainment and Enrollment eFigure 2. Sample Distribution of Susceptibility Across the Caudate, Putamen, and Globus Pallidum, in Female and Male Adolescents eFigure 3. Overall Results of the Partial Least Squares Analyses Examining the Associations Between Markers of Body Iron Status and Subcortical Structure Volumes, Psychopathology, and Neuropsychological Task Performance eFigure 4. Overall Results of the Partial Least Squares Analyses Examining the Associations Between Basal Ganglia Susceptibility Values and Subcortical Structure Volumes, Psychopathology, and Neuropsychological Task Performance eReferences. [file jamanetwopen-e2516687-s001.pdf]

Supplemental Online Content

Fiani D, Kim J, Hu M, et al. Iron deficiency without anemia and reduced basal ganglia iron content in youths. *JAMA Netw Open.* 2025;8(6):e2516687. doi:10.1001/jamanetworkopen.2025.16687

eMethods.

eResults.

eTable 1. Additional Clinical Characteristics of the Participants as a Group and Divided Based on Iron Deficiency Status

eTable 2. Results of the Multivariable Regression Analyses Examining the Associations Between Age, Iron Deficiency Status, and Their Interaction and Basal Ganglia Susceptibility

eTable 3. Strength of the Associations Between Basal Ganglia Susceptibility Values and Structural and Cognitive Outcomes Emerging from the Partial Least Squares Analyses

eFigure 1. Diagram Describing Participant Ascertainment and Enrollment

eFigure 2. Sample Distribution of Susceptibility Across the Caudate, Putamen, and Globus Pallidum, in Female and Male Adolescents

eFigure 3. Overall Results of the Partial Least Squares Analyses Examining the Associations Between Markers of Body Iron Status and Subcortical Structure Volumes, Psychopathology, and Neuropsychological Task Performance

eFigure 4. Overall Results of the Partial Least Squares Analyses Examining the Associations Between Basal Ganglia Susceptibility Values and Subcortical Structure Volumes, Psychopathology, and Neuropsychological Task Performance

eReferences.

This supplemental material has been provided by the authors to give readers additional information about their work.

© 2025 Fiani D et al. *JAMA Network Open*.

eMethods

Inclusion criteria:

1- Age 10 to 17 years.

2- Unmedicated.

3- Either having a mood and/or anxiety disorder (i.e., major depressive disorder, persistent depressive disorder, generalized anxiety disorder, social anxiety disorder, or separation anxiety disorder) or having no psychopathology.

Exclusion criteria:

1- Serious general medical condition, other than a mood or anxiety disorder.

2- Pregnancy.

3- Anemia, defined as having a hemoglobin concentration < 11.3 or < 11.2 g/dL for males and females younger than 13 years of age, respectively, and a hemoglobin concentration < 12.4 or < 11.4 g/dL, in those 13 years and older, respectively.[1]

4- Underweight (body mass index < 5th percentile) or malnourished.

5- Primary psychiatric disorder, requiring treatment, other than mood or anxiety disorders

6- Treatment with psychotropics within the prior 12 months

7- Presence of illicit drug and/or alcohol use disorders.

8- History of conditions (e.g., brain injury) or medication use (e.g., extended oral corticosteroids) associated with structural brain abnormality.

9- History of prematurity, macrosomia, exclusive breastfeeding for more than six months, or anemia (i.e., hemoglobin < 11.0 g/dL) before age 5 years.

10- Learning disorders.

11- Contraindication to undergoing brain MRI scan or wearing braces.

12- Inability to provide consent or assent.

© 2025 Fiani D et al. *JAMA Network Open*.

Most of these criteria sought to exclude participants with confounding conditions, including a history of perinatal iron deficiency. Confirming eligibility was first done based on medical record review followed by querying the parent/guardian.

Psychiatric Symptoms Assessment:

At the visit, the participants completed the Center for Epidemiological Studies Depression Scale for Children (CESD-C) and the Screen for Child Anxiety-Related Disorders (SCARED).[2,3] One parent/guardian completed the SCARED and the Child Behavior Checklist (CBCL 6-18 years).[4] A child and adolescent psychiatrist interviewed the participant and parent/guardian, separately, reviewed the participants' forms to establish the psychiatric diagnoses,[5] and completed the Children's Depression Rating Scale-Revised (CDRS-R) and the Pediatric Anxiety Rating Scale (PARS).[6,7] Race and ethnicity were self-reported. They were assessed given the difference in the prevalence of ID across racial and ethnic groups.[14]

Neuropsychological Tests:

1) Continuous Performance Test (CPT-III), a measure of response inhibition, sustained attention, vigilance, and signal detection.[8] The following outcomes were generated: T score of the number of hits, omission errors, commission errors, and d prime.

2) Vocabulary and Matrix Reasoning subtests from the Wechsler Abbreviated Scale of Intelligence – II, to capture overall intellectual functioning.[9]

3) Rey Auditory Verbal Learning Test, a word list repetition learning and memory task.[10-13] The following outcomes were generated: List A and list B trial 1 immediate recall, list A trials 1 and 2 of delayed recall, list A correct recognition, standard total learning (i.e., sum of the number of correct responses list A trials 1 through 5), learning ratio (i.e., total amount of new words learned

© 2025 Fiani D et al. *JAMA Network Open*.

after list A trial 1 divided by the number of remaining words not reported at trial 1), T1-T5 difference (i.e., correct words at list A trial 5 minus correct words at list A trial 1), early learning (i.e., average of list A trials 1 and 2), late learning (i.e., average of list A trials 4 and 5), proactive and retroactive interference (i.e., list B immediate recall minus list A trial 1 immediate recall and list A trial 1 delayed recall minus list A trial 5 immediate recall, respectively), retention (i.e., list A trial 2 delayed recall minus list A trial 5 immediate recall), and retrieval efficiency (i.e., list A correct recognition minus list A trial 5 immediate recall).[10]

4) Stroop Color and Word Test, a measure of response inhibition, selective attention, and perceptual set shifting.[14] The total number of words in black ink read in 45 seconds, total number of ink colors recognized in 45 seconds, and total number of ink colors correctly recognized despite incongruence with the word in 45 seconds were generated.

5) Trail Making A and B tests, which are measures of cognitive flexibility, set shifting, sequencing ability, visual-motor tracking, and visual-spatial functioning.[15] The following outcomes were generated: total time to complete Trails A and B, total number of sequencing errors for Trails A and B, and total number of set errors for Trail B.

6) Grooved Pegboard test, measuring manual dexterity and eye-hand coordination.[16] Performance for the total time to complete the task and for the total number of pegs dropped, respectively, were averaged for the right and left hand.

7) Forward, backward, and sequencing subtests of the Digit Span from the Wechsler Intelligence Scale for Children-Fourth Edition, a measure of working memory and simple auditory attention.[17]

8) Delay discounting task, a measure of one's ability to conceptualize future gains and update their values given delays in receiving them.[18] K value was generated.

Anthropometry

© 2025 Fiani D et al. *JAMA Network Open*.

Height was measured to the nearest 0.1 cm with a wall-mounted stadiometer (Ayrton Model S100, Hamburg, Germany) and weight was recorded to the nearest 0.1 kg (Seca 220 digital scale, Hamburg, Germany) with participants in indoor clothes without shoes. The average of two measurements was obtained. Body mass index (BMI) was computed as weight/height^2 (kg/m^2) and age-sex-specific BMI Z-scores were generated based on the 2000 Centers for Disease Control and Prevention normative data.[19]

Brain Imaging

<u>MRI Acquisition:</u> Imaging data were acquired on a 3T scanner (Achieva, Philips, Amsterdam, Netherlands). The first 48 (23%) scans were collected using a 15-channel receive-only head-neck coil, with the remainder collected using a 32-channel coil.

Quantitative susceptibility mapping (QSM) data were acquired with a 3D GRE multi-echo sequence in axial orientation, repetition time (TR) = 30.4 ms, 4 monopolar echoes of initial echo time (TE)1= 7ms with equal spacing of 7 ms, flow compensation for the first echo, flip angle (FA) = 10°, bandwidth = 649 Hz/pixel, covering a field of view (FOV) of 240 mm × 240 mm × 150 mm, voxel resolution 0.7 mm × 0.7 mm × 1.0 mm, with sensitivity encoding (SENSE) acceleration factor = 2 (phase-encoding) × 1.6 (slice), and acquisition time = 7.5 mins.

T1-weighted (T1w) 1.0 mm isotropic structural images were acquired using a 3D fast field echo (FFE) sequence, covering a FOV of 256 mm × 256 mm × 180 mm, TR/TE = 8.0/3.7 ms, FA = 6°, bandwidth = 192 Hz/pixel, SESNE = 2.5 (slice), and acquisition time = 5 mins 5 secs.

<u>QSM Reconstruction:</u> QSM reconstruction was performed using the Multi-Scale Dipole Inversion (MSDI) algorithm implemented in QSMbox v1.0.[20]

A three-step preprocessing was applied to compute brain mask for QSM reconstruction. First, using the T1w image, an initial mask was created using FSL's (v6.0.6.1) Brain Extraction Tool (BET) with an empirically defined fractional intensity threshold (-f) of 0.4.[21] This was followed by

© 2025 Fiani D et al. *JAMA Network Open*.

a phase variability (PV) map using UKBiobank's script on echo-weighted and combined phase image with an empirically defined voxel PV threshold > 0.90.[22] The final mask for QSM was created by filling a 5-time-eroded initial brain mask to avoid holes inside of the thresholded PV mask.[23]

Local field was estimated by phase unwrapping using ROMEO (v0.4.1) and magnitude-weighted least squares phase echo fitting on the multi-echo data.[24] Background field was removed using Laplacian Boundary Value (LBV) method followed by variable Spherical Mean Value (vSMV) algorithm with an initial kernel radius of 40 mm.[20,25] MSDI inversion was estimated at four scales: 2, 4, 8, 16 mm with default regularization term[20] and filtering was done with a kernel radius set to 5mm.

Susceptibility values are expressed in parts per billion (ppb) and referenced to the average susceptibility in the final QSM brain mask (i.e., average whole brain QSM value was subtracted from each individual QSM map).

QSM-T1w hybrid image and template: T1w structural image was registered to the first echo magnitude image of the QSM data and combined with the calculated QSM map to form a QSM-T1w hybrid image to take advantage of the enhanced subcortical QSM contrast.[26] The resulting QSM-T1w hybrid images from 111 representative participants from the study were used for normalization to form a study-specific QSM-T1w hybrid template using ANTs (v2.5.1).[27]

QSM regions of interest (ROIs): Three basal ganglia (BG) structures: caudate (Cd), putamen (Pu), and globus pallidus (GP) were manually defined in each hemisphere in our study specific QSM-T1w hybrid template. Of note, two subregions were purposefully excluded from the ROIs: (i) a small ventral corner of the Cd, with prominent veins and (ii) the medial side of the Pu, which contains myelinated striato-pallidal fibers (i.e., Wilson’s pencils).

© 2025 Fiani D et al. *JAMA Network Open*.

QSM ROI registration: Each ROI in the template space was transformed to the native QSM image space by combining the following registration steps, transformation type and degree of freedom (DOF) or non-linear algorithm in parentheses, using ANTs (v2.5.1): (i) QSM first echo magnitude image to T1w (affine, DOF12); (ii) T1w to AC-PC-aligned orientation (rigid, DOF6). The hybrid QSM-T1w image was generated in this AC-PC aligned orientation; (iii) AC-PC-aligned to our study specific template (whole brain) (nonlinear, SyN); (iv) constrained template to template (BG) (nonlinear, SyN), with a tight BG mask manually defined in our study specific template to spatially constrain the cost function and hence improve the performance of the nonlinear registration of the ROIs. After combining the transformations from the above steps, each ROI in our study-specific template was transformed to the QSM native space using one-step resampling with linear interpolation and then binarized using a threshold of 0.6.

Structural Image Analysis: T1w images were segmented using Freesurfer (v6.0.0) recon-all pipeline to generate the volumes of the segmented subcortical (-subcortseg) structures in both hemispheres: caudate, putamen, pallidum, amygdala, hippocampus, thalamus, and nucleus accumbens, as well as the estimated total intracranial volume (eTIV).[28]

Partial Least Squares Analysis:

Correlational partial least squares (PLS) analyses decompose the covariance matrix between two mean-centered datasets and identify the latent variable representing the impact of predictor variables (e.g., susceptibility value) on outcome variables (e.g., brain structures volume). This multivariate approach simultaneously captures the relationships between multiple predictors and outcome variables in a single model while effectively handling high multicollinearity, which is a common concern in high-dimensional data with intrinsically correlated variables, such as spatially correlated brain structures[29-31] or psychological constructs.[32-35] The analyses were run separately for males and females. To account for age effect, all the variables were adjusted for age before

© 2025 Fiani D et al. *JAMA Network Open*.

inclusion in the corresponding models. Moreover, subcortical volumetric variables were also adjusted for eTIV.

For each latent variable identified in PLS, the proportion of cross-block covariance explained — akin to r^2 and used as a measure of effect size — is calculated as the ratio of the squared *i*-th singular value to the total sum of all squared singular values. To assess the statistical significance of the PLS models, 10,000 permutations of the rows of the predictor variables were performed, and the observed cross-block covariance was compared to permuted cross-block covariances.[31,35] The stability of the left and right singular vectors was evaluated by 10,000 bootstrap resampling of both predictor and dependent variables matrices.[31,35] The bootstrap ratios were estimated by dividing the empirical loading by the bootstrap variance and were distributed normally under the null hypothesis. In other words, absolute bootstrap ratios approximate the units of standard error from the mean (i.e., akin to a T-score or Z-score). Then, two-tailed *p* values were computed for each bootstrap ratio and adjusted for multiple comparisons using Benjamini-Hochberg correction. Only variables with significant absolute bootstrap ratios were considered statistically significant.

Correlational PLS analyses used procedures from MATLAB2024 for Windows (The MathWorks Inc., Natick, MA).

© 2025 Fiani D et al. *JAMA Network Open*.

eResults

Of the 240 participants enrolled, 26 were excluded for neither having a mood or anxiety disorder nor being a healthy control (n=11), failure to comply with study procedures (n=5), having anemia (n=4), inability to complete the MRI scan (n=2), and having an eating disorder (n=1), underweight (n=1), macrosomia (n=1), or recent oral corticosteroid use (n=1). Another four participants had poor-quality MRI scans.

We conducted one set of PLS analyses to identify latent variables representing the effects of body iron, as captured by sF and hemoglobin concentration, on brain structures volume, psychopathology, and neuropsychological performance. A second set of PLS analyses examined the associations between caudate, putamen, and pallidum susceptibility, an index of their iron content, and the same outcome variables. The analyses were performed separately for males and females. Brain volumes for the right and left caudate, putamen, pallidum, thalamus, hippocampus, amygdala, and nucleus accumbens in addition to the eTIV were included. All these volumetric variables were adjusted for age and eTIV, except the latter, which was only adjusted for age. Psychopathology was captured with the Children's Depression Rating Scale – Revised (CDRSR) total score, the Pediatric Anxiety Rating Scale (PARS) score, the child-completed Center for Epidemiological Studies Depression Scale for Children (CESD-C) and the Screen for Child Anxiety-Related Disorders (SCARED), and the parent-completed SCARED and Child Behavior Checklist (CBCL 6-18 years). From the latter, the following scale scores were used: anxious-depressed, withdrawn-depressed, somatic complaints, social problems, thought problems, attention problems, rule-breaking behavior, aggressive behavior, activities, social functioning, and educational performance. All the raw scores were adjusted for age. In addition, the three items where higher score is associated with higher functioning (i.e., activities, social functioning, and educational performance) were reverse coded. Finally, 34 outcomes from the 8

© 2025 Fiani D et al. *JAMA Network Open*.

neuropsychological tests listed above were examined, after scores were adjusted for age. Of note, 149 participants had valid data on all neuropsychological tests (eTable 1) and were included in the PLS analyses examining the associations between iron content and neuropsychological performance. In addition, the k value from the delay discounting task was log-transformed.

For males, PLS did not find any significant latent variable for the effects of body iron on brain structure (p = .663, cross-block covariance = 62.12%), psychopathology (p = .198, cross-block covariance = 76.28%), or neuropsychological performance (p = .176, cross-block covariance = 71.49%). Similarly in females, PLS failed to capture any significant latent variable representing the effects of body iron on brain structure (p = .058, cross-block covariance = 72.58%), psychopathology (p = .110, cross-block covariance = 87.48%), or neuropsychological performance (p = .068, cross-block covariance = 64.23%). These results suggest that body iron content, as represented by sF and hemoglobin concentration, does not have a direct impact on the brain structures examined, psychopathology, or neuropsychological abilities in either male or female participants (eFigure 3).

Regarding the effects of basal ganglia susceptibility, PLS results for males revealed no significant latent variables for brain structure (p = .096, cross-block covariance = 54.56%), psychopathology (p = .075, cross-block covariance = 77.81%), or neuropsychological performance (p = .186, cross-block covariance = 42.59%). In contrast, in females, PLS identified a significant latent variable for the effect of basal ganglia susceptibility on brain structure (p < .001, cross-block covariance = 70.29%). In fact, susceptibility values in all basal ganglia structures considered significantly predicted brain structures volume (eTable 3). Specifically, higher susceptibility values were associated positively with the volumes of the left and right thalamus, left pallidum, left hippocampus, and eTIV and negatively with the left putamen and left nucleus accumbens. On the other hand, there were no significant latent variables for psychopathology (p = .319, cross-block covariance = 49.18%). Finally, PLS for the effect of basal ganglia susceptibility on

© 2025 Fiani D et al. *JAMA Network Open*.

neuropsychological performance identified a significant latent variable in females (p = .008, cross-block covariance = 57.57%), whereby susceptibility values in the putamen and caudate bilaterally were positively correlated with WASI vocabulary, correct recall rates in the RAVLT, Stroop task performance, and reduced errors in the Trail Making task (eFigure 4).

Together, these findings suggest that body iron does not appear to directly impact the volume of the brain structures examined, psychopathology, or neuropsychological functioning in either males or females. Nonetheless, female participants appear to be more affected by their body iron levels, as PLS analyses captured numerically greater covariance between body iron and downstream outcomes for them compared to males, and identified significant latent variables for the effects of basal ganglia susceptibility. Of note, higher basal ganglia susceptibility was associated with more adaptive outcomes, across the volumetric and neuropsychological domains.

© 2025 Fiani D et al. *JAMA Network Open*.

**eTable 1:** Additional Clinical Characteristics of the Participants as a Group and Divided Based on Iron Deficiency Status.				
	Total N=209	IDWoA N=62	No ID N=147	P value
**Psychiatric Rating Scales**				
CESD-C, mean (SD)	13.2±10.7	14.0±10.1	12.9±10.9	0.49
SCARED-Child, mean (SD)	23.9±16.7	27.9±17.4	22.3±16.1	**<0.03**
SCARED-Parent, mean (SD)	13.0±13.6	15.4±14.4	11.9±13.2	***<0.09***
CBCL, T-score, mean (SD)				
Anxious/Depressed	54.7±7.6	55.7±7.5	54.3±7.6	0.24
Withdrawn/Depressed	54.3±6.6	53.3±4.7	54.7±7.2	0.11
Somatic Complaints	55.6±6.9	55.5±6.9	55.9±6.9	0.72
Social Problems	52.2±4.1	52.7±4.3	52.0±4.1	0.26
Thought Problems	53.6±6.0	54.3±5.6	53.3±6.1	0.27
Attention Problems	52.6±4.5	53.2±4.6	52.3±4.4	0.22
Rule Breaking Behavior	51.2±2.2	51.1±2.0	51.2±2.3	0.90
Aggressive Behavior	51.1±2.6	51.0±2.2	51.2±2.7	0.66
Activities	45.5±10.4	45.7±10.8	45.5±10.3	0.86
Social Functioning	47.4±10.0	47.5±9.7	47.4±10.1	0.93
Educational Performance	52.1±4.8	52.0±4.8	52.1±5.0	0.85
**Neuropsychological data**				
CPT, mean (SD)				
Commission Error	38.0±18.5	35.4±17.0	39.0±19.0	0.20
Omission Error	2.4±3.8	2.6±4.6	2.3±3.5	0.63
Hits	280.0±12.7	279.4±15.1	280.3±11.6	0.68
d prime	-2.50±0.93	-2.60±0.93	-2.46±0.92	0.35
RAVLT List A[1], mean (SD)				
Immediate Recall T1	6.2±1.5	6.0±1.6	6.3±1.5	0.210.39
Correct Recognition	14.1±1.4	13.9±1.8	14.2±1.2	0.20
Delayed Recall T1	11.1±2.5	10.7±2.5	11.2±2.5	0.21
Delayed Recall T2	11.0±2.5	10.6±2.8	11.1±2.6	0.76
T1-T5 Difference	6.1±2.0	6.1±2.1	6.2±2.0	
RAVLT List B[1], mean (SD)				
Immediate Recall T1	5.6±1.6	5.4±1.6	5.7±1.6	0.30
RAVLT[1], mean (SD)				
Standard Total Learning	50.3±8.0	49.4±9.1	50.7±7.5	0.31
Learning Ratio	0.77±0.33	0.73±0.32	0.78±0.33	0.26
Early Learning	7.6±1.5	7.3±1.6	7.7±1.5	0.11
Late Learning	12.2±1.9	12.0±2.0	12.3±1.8	0.33
Proactive Interference	-0.63±1.87	-0.60±1.68	-0.64±1.94	0.89
Retroactive Interference	-1.30±1.67	-1.37±1.40	-1.27±1.77	0.69
Retention	-1.41±1.76	-1.49±1.62	-1.38±1.81	0.68
Retrieval Efficiency	1.73±1.76	1.86±1.88	1.68±1.71	0.51
Stroop[2], mean (SD)				
Word Read	85.5±16.7	84.2±16.7	86.0±16.7	0.50
Color Recognized	64.2±13.3	62.4±12.7	64.9±13.5	0.24
Ink Recognized	39.1±10.4	37.7±9.8	39.7±10.7	0.25
Trail Making Test A[3], mean (SD)	15.1±6.4	15.2±6.1	15.0±6.6	0.90

© 2025 Fiani D et al. *JAMA Network Open*.

Time<br>Sequencing Error	0.3±0.6	0.3±0.6	0.3±0.6	0.57
Trail Making Test – B[4], mean (SD)<br>Time<br>Sequencing Error<br>Set Error	35.4±24.1<br>0.3±0.<br>0.4±0.8	37.4±27.2<br>0.3±0.8<br>0.5±0.9	34.6±22.9<br>0.3±0.6<br>0.4±0.8	0.47<br>0.76<br>0.40
WASI (T-score)[5], mean (SD)<br>Vocabulary<br>Matrix Reasoning	49.3±8.3<br>52.6±9.7	***47.5±8.3***<br>***50.7±9.5***	***49.9±8.2***<br>***53.4±9.6***	***<0.06***<br>***<0.07***
Pegboard Right Hand[6], mean (SD)<br>Time, sec<br>Pegs Dropped	68.1±13.4<br>0.3±0.5	67.3±10.7<br>0.2±0.4	68.5±14.4<br>0.3±0.5	0.55<br>0.70
Pegboard Left Hand[6], mean (SD)<br>Time, sec<br>Pegs Dropped	74.3±14.6<br>0.4±0.6	73.7±14.3<br>0.3±0.5	74.6±14.8<br>0.4±0.7	0.70<br>0.27
Digit Span[7], mean (SD)<br>Forward<br>Backward<br>Sequencing	9.2±2.2<br>10.2±2.9<br>10.5±3.4	9.0±1.9<br>10.2±2.8<br>10.5±3.4	9.2±2.3<br>10.2±2.9<br>10.5±3.4	0.60<br>0.95<br>0.57
Delay Discounting[8]<br>k value, Median (Q1-Q3)	0.013<br>(0.005-0.049)	0.013<br>(0.005-0.040)	0.013<br>(0.005-0.058)	0.98

IDWoA: iron deficiency (defined as serum ferritin concentration <15 ng/mL) without anemia. No ID: serum ferritin concentration ≥15 ng/mL.
CESD-C: Center for Epidemiological Studies Depression Scale for Children. SCARED: Screen for Child Anxiety Related Disorders. CBCL: Child Behavior Checklist (6-18yrs). CPT: Continuous Performance Test III. RAVLT: Rey Auditory Verbal Learning Test. WASI: Wechsler Abbreviated Scale of Intelligence – II.
1: Only 200 participants (57 with IDWoA) had valid RAVLT data.
2: Only 196 participants (55 with IDWoA) had valid Stroop Color and Word Test data.
3: Only 192 participants (53 with IDWoA) had valid Trail Making Test – A data.
4: Only 194 participants (54 with IDWoA) had valid Trail Making Test – B data.
5: Only 205 participants (61 with IDWoA) had valid data for the vocabulary and matrix reasoning subtests of the Wechsler Abbreviated Scale of Intelligence – II.
6: Only 197 participants (56 with IDWoA) had valid Grooved Pegboard test data for the right hand and 195 (55 with IDWoA) had valid data for the left hand.
7: Only 206 participants (60 with IDWoA) had valid data on the forward substest of the digit span, 197 (59 with IDWoA) on the backward subtest, and 201 (59 with IDWoA) on the sequencing subtest.
8: Only 204 participants (58 with IDWoA) had valid delay discounting task data. Given the skewed distribution, the Wilcoxon rank sum test compared the performance between the two groups.
Marginally significant (0.05<p<0.10) results are bolded and italicized.

© 2025 Fiani D et al. *JAMA Network Open*.

eTable 2: Results of the multivariable regression analyses examining the associations between age, iron deficiency status, and their interaction and basal ganglia susceptibility

	Caudate (ppb)		Putamen (ppb)		Pallidum (ppb)	
	Females	Males	Females	Males	Females	Males
Age, years	0.20 (-0.60 – 1.00)	-0.29 (-1.86 – 1.28)	**0.81 (0.22 – 1.40)**	1.31 (-0.08 – 2.70)	**2.05 (0.26 – 3.84)**	0.37 (-3.89 – 4.64)
IDWoA	-11.54 (-25.69 – 2.61)	-12.40 (-34.71 – 9.91)	**-10.75 (-21.21 – -0.29)**	3.83 (-15.91 – 23.56)	-12.60 (-44.45 – 19.25)	-43.79 (-104.31 – 16.74)
Interaction	**1.11 (0.08 – 2.15)**	0.93 (-0.72 – 2.58)	**0.95 (0.18 – 1.71)**	-0.20 (-1.66 – 1.26)	1.01 (-1.32 – 3.33)	3.19 (-1.29 – 7.67)

IDWoA: iron deficiency (defined as serum ferritin concentration <15 ng/mL) without anemia.
Regression estimates along with their 95% confidence interval. Participants with IDWoA comprised the reference group.
Significant results (p<0.05) are bolded.

© 2025 Fiani D et al. *JAMA Network Open*.

eTable 3: Strength of the Associations Between Basal Ganglia Susceptibility Values and Structural and Cognitive Outcomes Emerging from the Partial Least Squares Analyses

	Region of Interest	Bootstrap ratio	$p_{adjusted}$
**Susceptibility ~ Brain Volume**	**Left Putamen**	**-2.6272**	**0.09**
	**Right Putamen**	**-5.0879**	**<.001**
	**Left Caudate**	**-4.5948**	**<.001**
	**Right Caudate**	**-4.9149**	**<.001**
	**Left Globus Pallidus**	**2.6255**	**<0.01**
	**Right Globus Pallidus**	**3.0171**	**<0.01**
**Susceptibility ~ Task Performance**	**Left Putamen**	**2.4631**	**0.03**
	**Right Putamen**	**2.3001**	**0.03**
	**Left Caudate**	**3.5381**	**0.001**
	**Right Caudate**	**4.6207**	**<.001**
	Left Globus Pallidus	-0.8282	0.49
	Right Globus Pallidus	-0.0424	0.97

Significant (p<0.05) results are bolded.

© 2025 Fiani D et al. *JAMA Network Open*.

eFigure 1: CONSORT diagram describing participant ascertainment and enrollment.

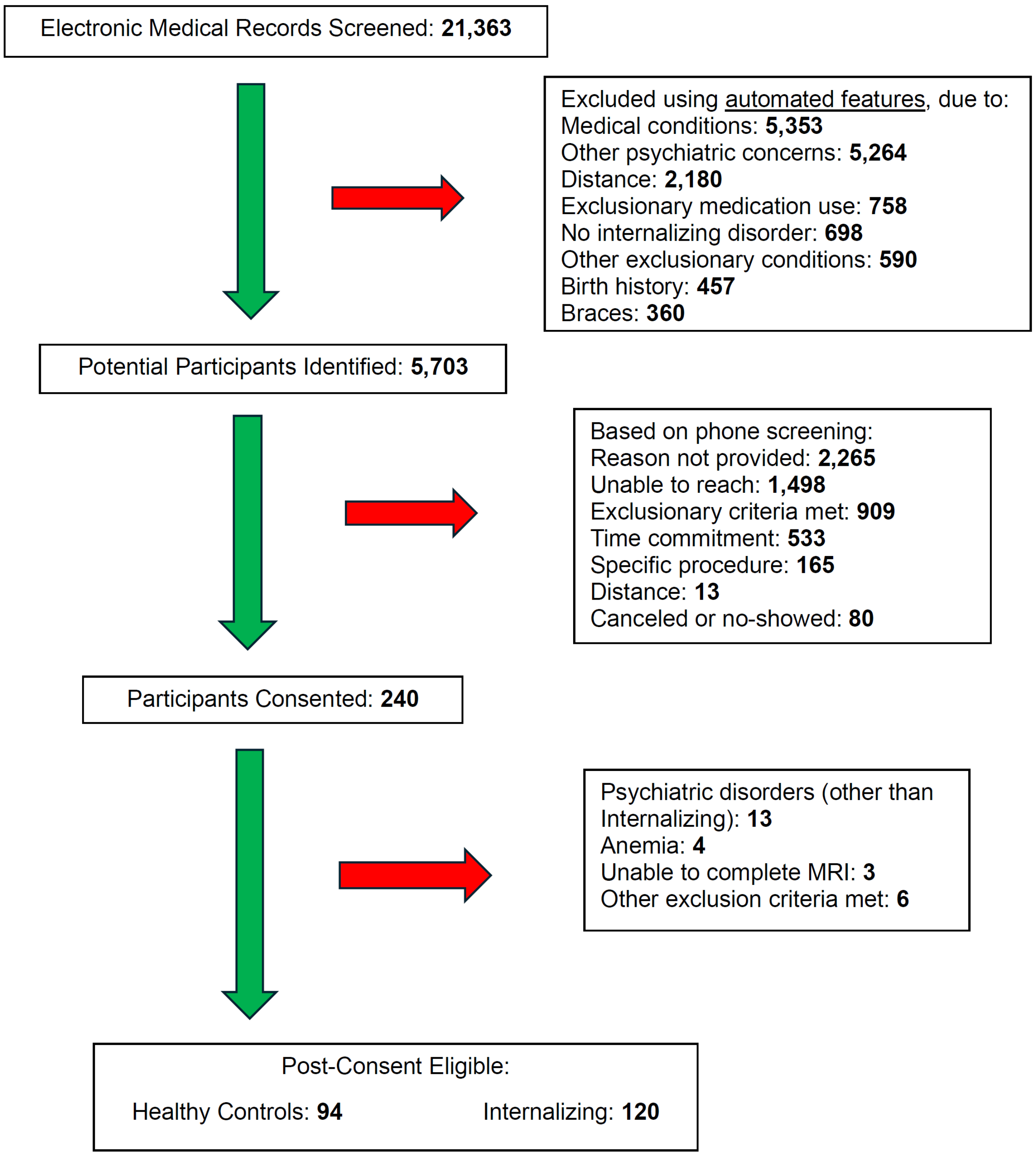

© 2025 Fiani D et al. *JAMA Network Open*.

eFigure 2: Sample distribution of susceptibility (parts per billion) across the caudate (Cd), putamen (Pu), and globus pallidum (GP), in female (panels A, C, and E) and male (panels B, D, and F) adolescents. Individual points represent susceptibility values for individual participants. Boxplots indicate the median value, along with the first and third quartiles of the sample distribution.

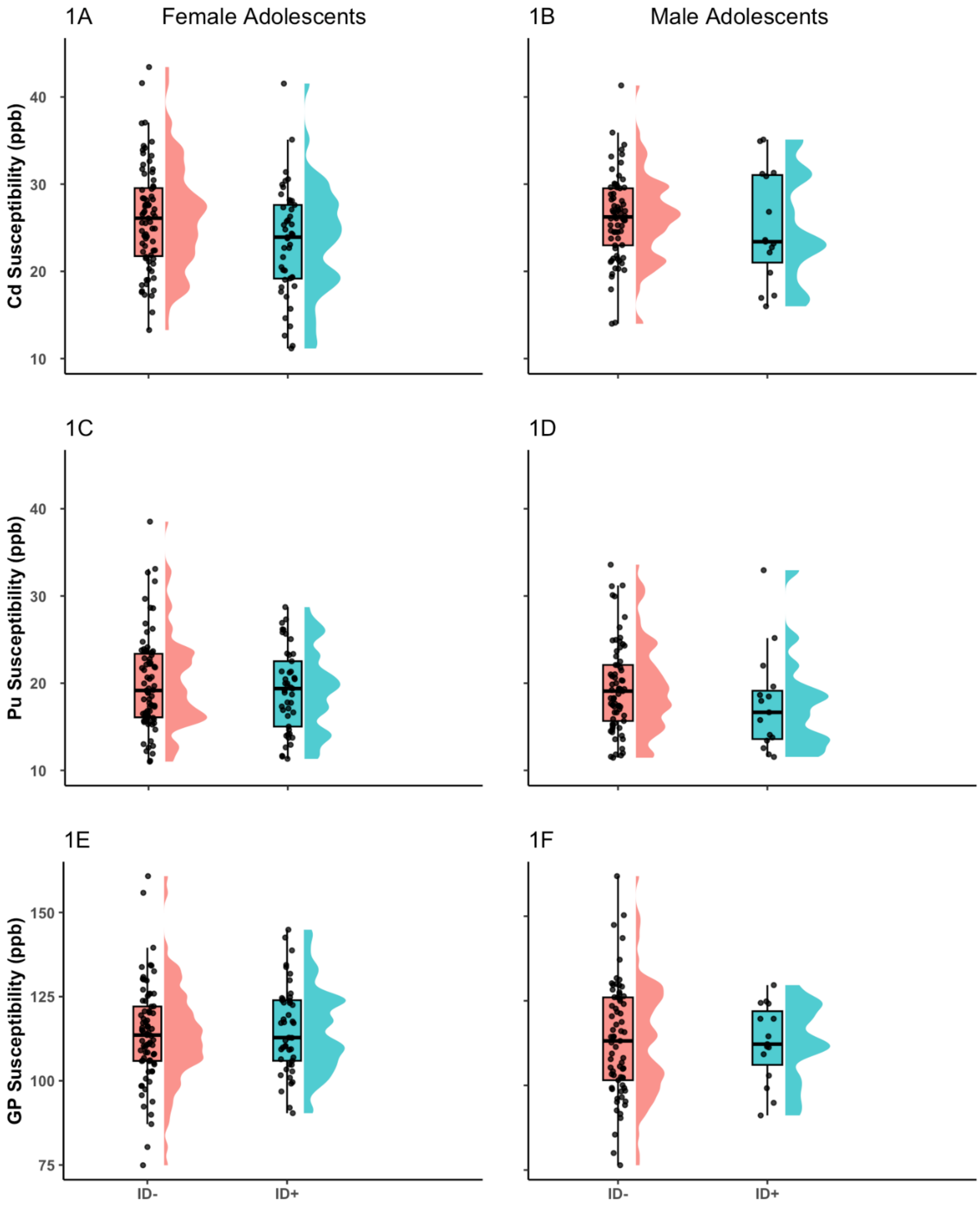

© 2025 Fiani D et al. *JAMA Network Open*.

eFigure 3. Overall Results of the Partial Least Squares Analyses Examining the Associations Between Markers of Body Iron Status (i.e., Serum Ferritin [ng/mL] and Hemoglobin (g/dL) Concentrations) and Subcortical Structure Volumes (mm^3), Psychopathology, and Neuropsychological Task Performance.

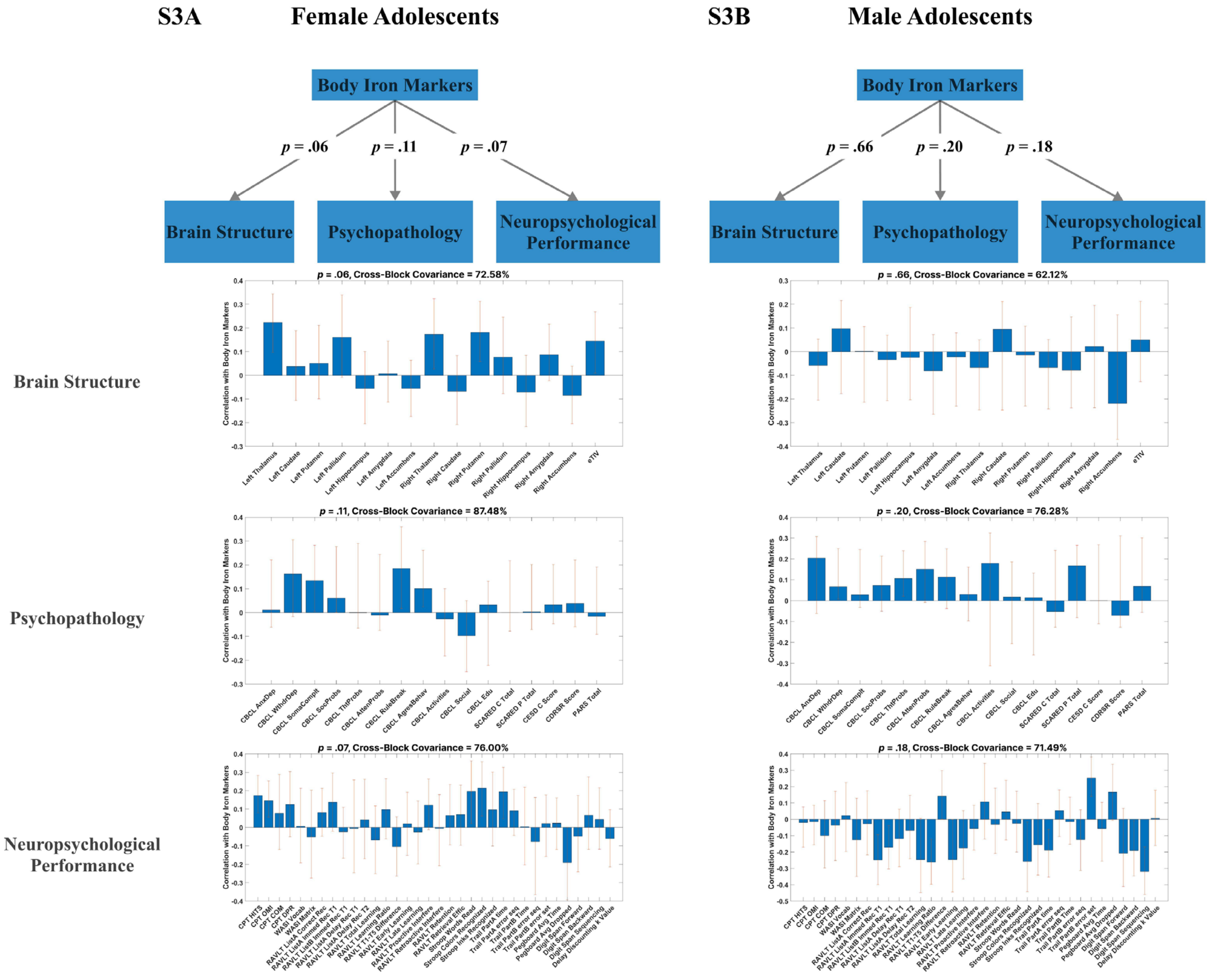

© 2025 Fiani D et al. *JAMA Network Open*.

The “Psychopathology” domain comprises the Anxious/Depressed, Withdrawn/Depressed, Somatic Complaints, Social Problems, Thought Problems, Attention Problems, Rule Breaking Behavior, Aggressive Behavior, Activities, Social Functioning, and Educational Performance subscales of the Child Behavior Checklist (CBCL), the Center for Epidemiological Studies Depression Scale for Children (CESD-C) and the Screen for Child Anxiety-Related Disorders (SCARED). The latter was completed by the participant and the parent, independently.
The “Neuropsychological” domain comprises the following test scores: Commission Error, Omission Error, Hits, and d prime from the Continuous Performance Test, 3rd edition, the Score on the Vocabulary and Matrix Reasoning subtests from the Wechsler Abbreviated Scale of Intelligence – II (WASI), List A Immediate Recall-Trial 1, List A Correct Recognition, List A Delayed Recall-T1 and T2, List A T1-T5 Difference, List B Immediate Recall-T1, Standard Total Learning, Learning Ratio, Early Learning, Late Learning, Proactive Interference, Retroactive Interference, Retention, and Retrieval Efficiency from the Rey Auditory Verbal Learning Test (RAVLT), the number of Words Read, Colors Recognized, and Inks Recognized from the Stroop Word and Color Test, Time to Complete and the number of Sequencing Errors from the Trail Making Test A, Time to complete and the number of Sequencing Errors and Set Errors from the Trail Making Test B, Time to complete and the number of Pegs Dropped combined for the right and left hand from the Grooved Pegboard test, the Forward, Backward, and Sequencing subtests of the Digit Span Test, and the k value from the Delay Discounting Task (DDT).
All variables were adjusted for age and the subcortical volume variables were also adjusted for Estimated total intracranial volume (eTIV). Models were run separately for female (panel A) and male (panel B) participants.
The bars reflect the Pearson's *r* correlation between the specific outcome variables and the overall concept represented by the predictors, as captured by their latent variable scores (i.e., an aggregated measure of serum ferritin and hemoglobin concentrations). Error bars crossing zero indicate non-significant correlations.

© 2025 Fiani D et al. *JAMA Network Open*.

eFigure 4. Overall Results of the Partial Least Squares Analyses Examining the Associations Between Basal Ganglia Susceptibility Values (parts per billion) and Subcortical Structure Volumes (mm³), Psychopathology, and Neuropsychological Task Performance.

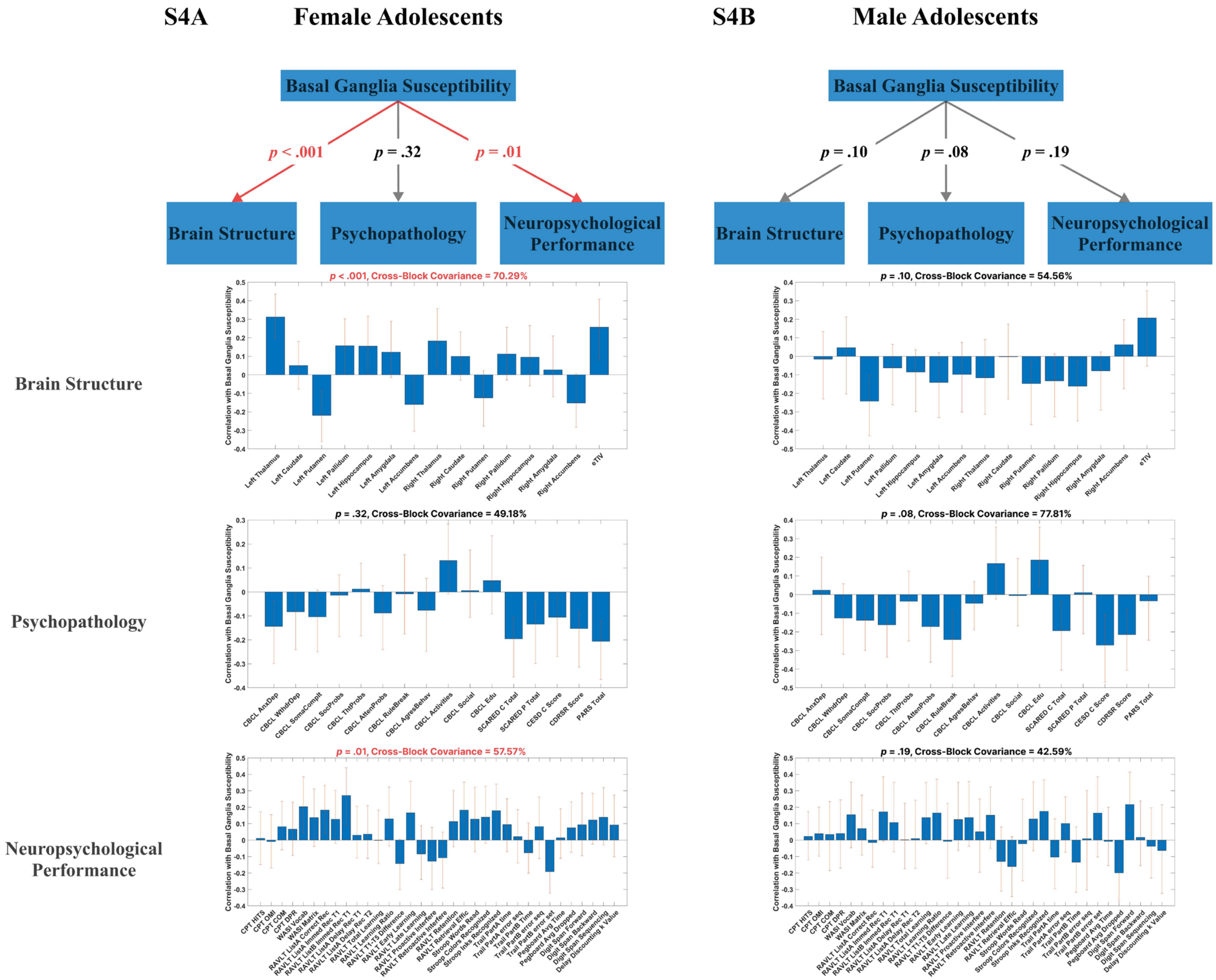

© 2025 Fiani D et al. *JAMA Network Open*.

The “Psychopathology” domain comprises the Anxious/Depressed, Withdrawn/Depressed, Somatic Complaints, Social Problems, Thought Problems, Attention Problems, Rule Breaking Behavior, Aggressive Behavior, Activities, Social Functioning, and Educational Performance subscales of the Child Behavior Checklist (CBCL), the Center for Epidemiological Studies Depression Scale for Children (CESD-C) and the Screen for Child Anxiety-Related Disorders (SCARED). The latter was completed by the participant and the parent, independently.
The “Neuropsychological” domain comprises the following test scores: Commission Error, Omission Error, Hits, and d prime from the Continuous Performance Test, 3rd edition, the Score on the Vocabulary and Matrix Reasoning subtests from the Wechsler Abbreviated Scale of Intelligence – II (WASI), List A Immediate Recall-Trial 1, List A Correct Recognition, List A Delayed Recall-T1 and T2, List A T1-T5 Difference, List B Immediate Recall-T1, Standard Total Learning, Learning Ratio, Early Learning, Late Learning, Proactive Interference, Retroactive Interference, Retention, and Retrieval Efficiency from the Rey Auditory Verbal Learning Test (RAVLT), the number of Words Read, Colors Recognized, and Inks Recognized from the Stroop Word and Color Test, Time to Complete and the number of Sequencing Errors from the Trail Making Test A, Time to complete and the number of Sequencing Errors and Set Errors from the Trail Making Test B, Time to complete and the number of Pegs Dropped combined for the right and left hand from the Grooved Pegboard test, the Forward, Backward, and Sequencing subtests of the Digit Span Test, and the k value from the Delay Discounting Task (DDT).
All variables were adjusted for age and the subcortical volume variables were also adjusted for Estimated total intracranial volume (eTIV). Models were run separately for female (panel A) and male (panel B) participants.
The bars reflect the Pearson's *r* correlation between the specific outcome variables and the overall concept represented by the predictors, as captured by their latent variable scores (i.e., an aggregated measure of the susceptibility values in the right and left caudate, putamen, and pallidum). Error bars crossing zero indicate non-significant correlations.

© 2025 Fiani D et al. *JAMA Network Open*.

eReferences

1. Staffa SJ, Joerger JD, Henry E, Christensen RD, Brugnara C, Zurakowski D. Pediatric hematology normal ranges derived from pediatric primary care patients. *Am J Hematol*. Oct 2020;95(10):E255-E257. doi:10.1002/ajh.25904
2. Birmaher B, Brent DA, Chiappetta L, Bridge J, Monga S, Baugher M. Psychometric properties of the Screen for Child Anxiety Related Emotional Disorders (SCARED): a replication study. *J Am Acad Child Adolesc Psychiatry*. Oct 1999;38(10):1230-6. doi:10.1097/00004583-199910000-00011
3. Faulstich ME, Carey MP, Ruggiero L, Enyart P, Gresham F. Assessment of depression in childhood and adolescence: an evaluation of the Center for Epidemiological Studies Depression Scale for Children (CES-DC). *Am J Psychiatry*. Aug 1986;143(8):1024-7. doi:10.1176/ajp.143.8.1024
4. Achenbach TM, Rescorla LA. *Manual for the ASEBA School-Age Froms & Profiles*. Research Center for Children, Youth & Families; 2001.
5. Association AP. *Diagnostic and Statistical Manual of Mental Disorders, Fifth Edition*. 5th ed. American Psychiatric Publishing; 2013.
6. The Pediatric Anxiety Rating Scale (PARS): development and psychometric properties. *J Am Acad Child Adolesc Psychiatry*. Sep 2002;41(9):1061-9. doi:10.1097/00004583-200209000-00006
7. Poznanski E, Mokros H. Psychometric properties of the CDRS-R. *Children's Depression Rating Scale, Revised (CDRS-R)*. Western Psychological Services; 2005.
8. Conners CK. *Conner's Continuous Performance Test 3rd Edition*. Multi-Health Systems Inc.; 2014.
9. Wechsler D. *Wechsler Abbreviated Scale of Intelligence Second Edition*. The Psychological Corporation; 2011.
10. Almkvist O, Rennie A, Westman E, Wallert J, Ekman U. Methods for assessment of rey auditory verbal learning test performance in memory clinic patients and healthy adults - at the cross-roads of learning theory and clinical utility. *Clin Neuropsychol*. Aug 12 2024:1-15. doi:10.1080/13854046.2024.2384616
11. Hammers DB, Spencer RJ, Apostolova LG, Alzheimer's Disease Neuroimaging I. Validation of and Demographically Adjusted Normative Data for the Learning Ratio Derived from the RAVLT in Robustly Intact Older Adults. *Arch Clin Neuropsychol*. Jul 19 2022;37(5):981-993. doi:10.1093/arclin/acac002
12. Vakil E, Greenstein Y, Blachstein H. Normative data for composite scores for children and adults derived from the Rey Auditory Verbal Learning Test. *Clin Neuropsychol*. May 2010;24(4):662-77. doi:10.1080/13854040903493522
13. Lezak MD, Howieson DB, Loring DW, Hannay HJ, Fischer JS. *Neuropsychological Assessment 4th Edition*. Oxford University Press; 2004.
14. Golden CJ, Freshwater SM, Golden Z. *Stroop, Color and Word Test, Children's Version for Ages 5-14*. Stoelting Co.; 2004.
15. Tombaugh TN. Trail Making Test A and B: normative data stratified by age and education. *Arch Clin Neuropsychol*. Mar 2004;19(2):203-14. doi:10.1016/S0887-6177(03)00039-8
16. Wang YC, Magasi SR, Bohannon RW, et al. Assessing dexterity function: a comparison of two alternatives for the NIH Toolbox. *J Hand Ther*. Oct-Dec 2011;24(4):313-20; quiz 321. doi:10.1016/j.jht.2011.05.001
17. Wechsler D. *Wechsler Intelligence Scale for Children-Fourth Edition (WISC-IV) administration and scoring manual*. The Psychological Corporation; 2003.
18. Frost R, McNaughton N. The neural basis of delay discounting: A review and preliminary model. *Neurosci Biobehav Rev*. Aug 2017;79:48-65. doi:10.1016/j.neubiorev.2017.04.022

© 2025 Fiani D et al. *JAMA Network Open*.

19. Ogden CL, Kuczmarski RJ, Flegal KM, et al. Centers for Disease Control and Prevention 2000 growth charts for the United States: improvements to the 1977 National Center for Health Statistics version. *Pediatrics*. Jan 2002;109(1):45-60.
20. Acosta-Cabronero J, Milovic C, Mattern H, Tejos C, Speck O, Callaghan MF. A robust multi-scale approach to quantitative susceptibility mapping. *Neuroimage*. Dec 2018;183:7-24. doi:10.1016/j.neuroimage.2018.07.065
21. Smith SM. Fast robust automated brain extraction. *Hum Brain Mapp*. Nov 2002;17(3):143-55. doi:10.1002/hbm.10062
22. Wang C, Martins-Bach AB, Alfaro-Almagro F, et al. Phenotypic and genetic associations of quantitative magnetic susceptibility in UK Biobank brain imaging. *Nat Neurosci*. Jun 2022;25(6):818-831. doi:10.1038/s41593-022-01074-w
23. Committee QSMCO, Bilgic B, Costagli M, et al. Recommended implementation of quantitative susceptibility mapping for clinical research in the brain: A consensus of the ISMRM electro-magnetic tissue properties study group. *Magn Reson Med*. May 2024;91(5):1834-1862. doi:10.1002/mrm.30006
24. Dymerska B, Eckstein K, Bachrata B, et al. Phase unwrapping with a rapid opensource minimum spanning tree algorithm (ROMEO). *Magn Reson Med*. Apr 2021;85(4):2294-2308. doi:10.1002/mrm.28563
25. Zhou D, Liu T, Spincemaille P, Wang Y. Background field removal by solving the Laplacian boundary value problem. *NMR Biomed*. Mar 2014;27(3):312-9. doi:10.1002/nbm.3064
26. Zhang Y, Wei H, Cronin MJ, He N, Yan F, Liu C. Longitudinal atlas for normative human brain development and aging over the lifespan using quantitative susceptibility mapping. *Neuroimage*. May 1 2018;171:176-189. doi:10.1016/j.neuroimage.2018.01.008
27. Tustison NJ, Cook PA, Holbrook AJ, et al. The ANTsX ecosystem for quantitative biological and medical imaging. *Sci Rep*. Apr 27 2021;11(1):9068. doi:10.1038/s41598-021-87564-6
28. Fischl B, Salat DH, Busa E, et al. Whole brain segmentation: automated labeling of neuroanatomical structures in the human brain. *Neuron*. Jan 31 2002;33(3):341-55. doi:10.1016/s0896-6273(02)00569-x
29. Krishnan A, Williams LJ, McIntosh AR, Abdi H. Partial Least Squares (PLS) methods for neuroimaging: a tutorial and review. *Neuroimage*. May 15 2011;56(2):455-75. doi:10.1016/j.neuroimage.2010.07.034
30. McIntosh AR, Lobaugh NJ. Partial least squares analysis of neuroimaging data: applications and advances. *Neuroimage*. 2004;23 Suppl 1:S250-63. doi:10.1016/j.neuroimage.2004.07.020
31. Stier AJ, Cardenas-Iniguez C, Kardan O, et al. A pattern of cognitive resource disruptions in childhood psychopathology. *Netw Neurosci*. 2023;7(3):1153-1180. doi:10.1162/netn_a_00322
32. Giessing C, Fink GR, Rosler F, Thiel CM. fMRI data predict individual differences of behavioral effects of nicotine: a partial least square analysis. *J Cogn Neurosci*. Apr 2007;19(4):658-70. doi:10.1162/jocn.2007.19.4.658
33. Ketterlinus RD, Bookstein FL, Sampson PD, Lamb ME. Partial least squares analysis in developmental psychopathology. *Development and Psychopathology*. 1989;1(4):351-371. doi:10.1017/S0954579400000523
34. Raffard S, Bortolon C, Burca M, Gely-Nargeot MC, Capdevielle D. Multidimensional model of apathy in older adults using partial least squares--path modeling. *Age (Dordr)*. Jun 2016;38(3):55. doi:10.1007/s11357-016-9916-z
35. Willaby HW, Costa DS, Burns BD, MacCann C, Roberts RD. Testing complex models with small sample sizes: A historical overview and empirical demonstration of what Partial Least Squares (PLS) can offer differential psychology. *Personality and Individual Differences*. 2015;84:73-78.

© 2025 Fiani D et al. *JAMA Network Open*.
