# Supplementary material for: Iron Deficiency Without Anemia and Reduced Basal Ganglia Iron Content in Youths
Source: JAMA Netw Open. 2025 Jun 20;8(6):e2516687. doi: 10.1001/jamanetworkopen.2025.16687 (PMC12181794; doi:10.1001/jamanetworkopen.2025.16687)
Supplement: Supplement 2. — Data Sharing Statement [file jamanetwopen-e2516687-s002.pdf]

Data Sharing Statement

Fiani. Iron Deficiency Without Anemia and Reduced Basal Ganglia Iron Content in Youths. *JAMA Netw Open*. Published June 20, 2025. doi:10.1001/jamanetworkopen.2025.16687

Data
Data available: Yes
Data types: Deidentified participant data
How to access data: The NIMH Data Archive: https://nda.nih.gov/ This is required by the funding agency.
When available: beginning date: 08/31/2026

Supporting Documents
Document types: None

Additional Information
Who can access the data: Anyone approved by the NIMH.
Types of analyses: No restrictions, except any placed by the funding agency. **Mechanisms of data availability:** Without investigator support. Access will be controlled by the NIMH.
Any additional restrictions: None.
